# Nasal Septal Perforation in Propylthiouracil-Induced Anti-Neutrophil Cytoplasmic Antibody-Associated Vasculitis

**DOI:** 10.1155/2018/8192021

**Published:** 2018-03-22

**Authors:** Yusho Ishii, Tsuyoshi Shirai, Yousuke Hoshi, Yoko Fujita, Yuko Shirota, Hiroshi Fujii, Tomonori Ishii, Hideo Harigae

**Affiliations:** Department of Hematology and Rheumatology, Tohoku University Graduate School of Medicine, Sendai, Japan

## Abstract

Here, we present the case of a 29-year-old woman with nasal septal perforation and positive myeloperoxidase- (MPO-) anti-neutrophil cytoplasmic antibody (ANCA). She had been diagnosed with Graves' disease and had been treated with propylthiouracil (PTU) for 14 months. A biopsy of the nasal septum revealed an infiltration of inflammatory cells, with no evidence of malignancy or granulomatous change. Because of the use of PTU, destructive nasal lesion, and positive MPO-ANCA, she was diagnosed with drug-induced ANCA-associated vasculitis (AAV) and was treated with prednisolone and methotrexate after the cessation of PTU. Although PTU is known to be the medicine that induces drug-induced AAV, the manifestation of nasal septal perforation in drug-induced AAV is poorly identified. This is the rare case of drug-induced AAV which manifested only nasal septal perforation.

## 1. Introduction

Anti-neutrophil cytoplasmic antibody- (ANCA-) associated vasculitis (AAV) generally occurs in middle-aged and older adults, and AAV patients manifest heterogeneous symptoms, such as fever, weight loss, myalgia, skin vasculitis, and sinonasal involvement. AAV involves the inflammation of small and medium arteries, which results in neuropathy, interstitial pneumonia, glomerulonephritis, otitis media, and sinusitis [1]. AAV comprises three different diseases: granulomatosis with polyangiitis (GPA), eosinophilic granulomatosis with polyangiitis, and microscopic polyangiitis [2]. In addition, some drugs cause drug-induced AAV [3]. In particular, propylthiouracil (PTU) and thiamazole, which are used to treat Graves' disease (GD), are known to induce ANCA and AAV [3, 4]. Here, we report the first case of PTU-induced AAV that manifested with only nasal septal perforation.

## 2. Case Presentation

**Figure 1:**
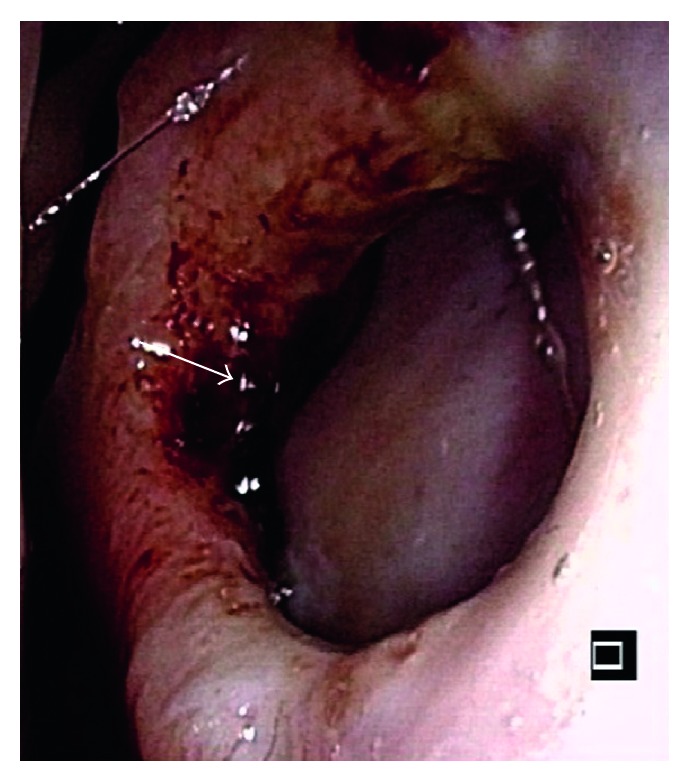
Nasal endoscopic finding. Nasal endoscopy shows nasal septal perforation.

**Figure 2:**
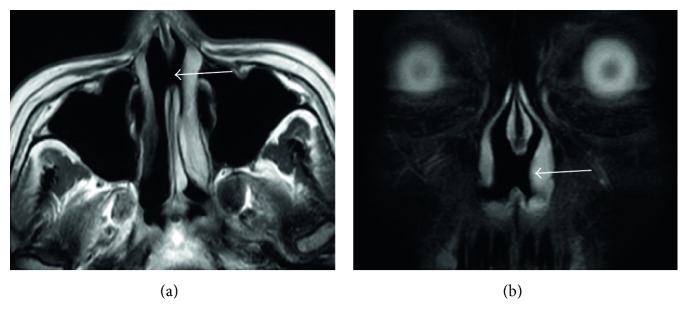
Magnetic resonance imaging (MRI) findings. MRI shows nasal septal perforation in horizontal (a) and coronal (b) sections.

**Figure 3:**
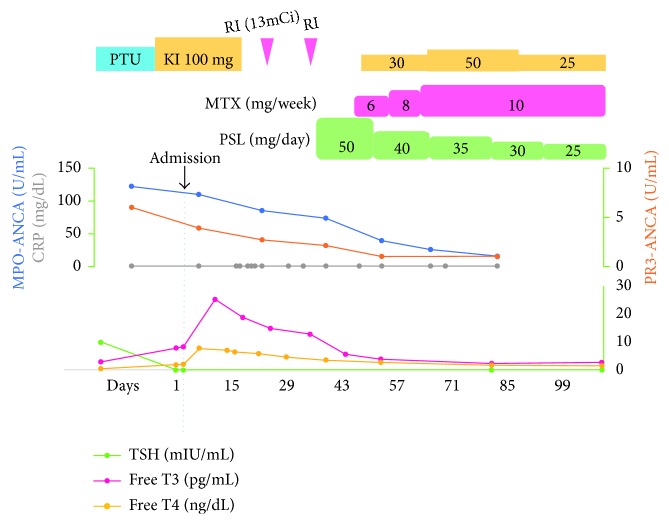
Clinical course. CRP: C-reactive protein; free T3: free triiodothyronine; free T4: free thyroxine; KI: potassium iodide; MPO-ANCA: myeloperoxidase-anti-neutrophil cytoplasmic antibody; MTX: methotrexate; PR3-ANCA: proteinase 3-anti-neutrophil cytoplasmic antibody; PSL: prednisolone; PTU: propylthiouracil; RI (13mCi): radioisotope (13mCi); TSH: thyroid-stimulating hormone.

A 29-year-old woman was referred to our hospital for the evaluation of nasal septal perforation with positive MPO-ANCA. Fifteen months before referral, she had been diagnosed with GD and consequently treated with PTU for 14 months. Two months before referral, she had developed a low-grade fever and sore throat and visited the otorhinolaryngology department. The otorhinolaryngologist detected nasal septal perforation and performed biopsy that revealed an infiltration of inflammatory cells into the basal layer of the epidermis, with no evidence of malignancy. In addition, she had never taken cocaine. AAV was suspected because MPO-ANCA was positive, and she was subsequently referred and admitted to our hospital. Because she was taking PTU and drug-induced AAV was suspected, PTU treatment was stopped a month before referral. On admission, thyroid gland swelling was detected. Laboratory findings, including those of urinalysis, blood biochemistry, and coagulation testing as well as complete blood count, were within the normal range. MPO-ANCA was positive (110 U/mL, 3.4 is a reference value in our hospital), and proteinase 3- (PR3-) ANCA was slightly positive (3.9 U/mL, 3.4 is a reference value in our hospital). Other autoantibodies were negative, except for anti-thyroid-stimulating hormone (TSH) receptor antibody (7.37 IU/L). Free thyroxine and free triiodothyronine levels were elevated, and the TSH level was decreased. Nasal endoscopy revealed nasal septal perforation (Figure 1). Computed tomography revealed the absence of sinusitis and pneumonia. Magnetic resonance imaging (MRI) revealed nasal septal perforation (Figure 2). We again performed a biopsy of the perforated nasal septum for differential diagnosis, which revealed an infiltration of inflammatory cells, including neutrophils and lymphocytes, with no evidence of malignancy or granulomatous change. Together with a destructive nasal lesion and positive MPO-ANCA, she was diagnosed with AAV, particularly PTU-induced AAV, based on the classification of vasculitis proposed by Watts et al. [5]. After the cessation of PTU, the titers of both MPO-ANCA and PR3-ANCA exhibited spontaneous reduction. Because the nasal septal perforation was destructive and had the potential to develop into a saddle nose, she desired treatment with high-dose prednisolone (1 mg/kg/day) and methotrexate (10 mg/week). She was treated with this regimen and discharged 1 month later. We are currently monitoring the nasal septal perforation by endoscopy and MRI, and progression has not yet been observed for 15 months. The titers of both MPO-ANCA and PR3-ANCA have been decreasing within our reference value (Figure 3).

## 3. Discussion

In this report, we present the case of a young woman who developed nasal septal perforation with a high titer of MPO-ANCA and a low titer of PR3-ANCA following the use of PTU for 14 months. ANCA is regarded as a marker of small vessel vasculitis. However, some patients who test positive for ANCA exhibit infections (such as tuberculosis), inflammatory bowel diseases, other autoimmune disorders, and malignancies [6–10]. Moreover, positivity for both MPO-ANCA and PR3-ANCA is reported to appear in subacute bacterial endocarditis [11]. PTU is prescribed for treating GD and is a medicine that induces drug-induced AAV. It is reported that 15%–64% of patients taking PTU exhibit ANCA positivity and that one-third to one-fourth of patients taking PTU present some symptoms of AAV [12]. Drug-induced AAV usually manifests with mild symptoms, including rash, arthralgia, myalgia, fever, fatigue, or anorexia. However, in some cases, severe symptoms, such as alveolar hemorrhage or rapid progressive glomerulonephritis, may appear [12].

The manifestation in our patient was limited to nasal septal perforation. The abnormal findings of the paranasal sinus are reported to appear in 85% of GPA patients, 25% of whom present only these symptoms [13]. Nasal septal perforation is found in approximately 11%–33% of GPA patients and is identified by epistaxis in most of them [13–16]. The chronic use of vasoconstrictive nasal sprays or cocaine may cause nasal septal perforation, and cocaine occasionally induces ANCA as well [17]. One case report presented a 26-year-old woman with PTU-induced AAV who manifested severe skin ulcer, pulmonary infiltrates, and nasal septal perforation [18]. To our knowledge, this is the only report presenting PTU-induced AAV with nasal septal perforation, which was observed in a patient with systemic involvement. Our case is different because her manifestation was limited to the nasal septal perforation. The prognosis of drug-induced AAV is better than that of primary AAV, and corticosteroid treatment can be steadily reduced and finally discontinued [19]. As demonstrated in this case, drug-induced AAV is characterized by onset at a younger age, positivity for both MPO-ANCA and PR3-ANCA, and better prognosis than that of primary AAV [12].

In conclusion, GD patients who are taking PTU are at a risk of developing AAV. Nasal septal perforation is rare but can be the only manifestation of drug-induced AAV.
